# Chronic psychosocial stress: a role in breast cancer etiology?

**DOI:** 10.3389/fonc.2026.1790548

**Published:** 2026-05-14

**Authors:** Roar Fosse, Cathrine Behr

**Affiliations:** 1 Department of Research and Development, Division of Mental Health and Addiction, Vestre Viken Hospital Trust, Drammen, Norway; 2 Private Practitioner, Asker, Norway

**Keywords:** breast cancer, chronic psychosocial stress, epidemiology, etiology, preclinical rodent research

## Abstract

**Background:**

Epidemiological and preclinical studies are discrepant regarding the role of psychosocial stress in breast cancer (BC) development and metastasis. While no consensus has been reached based on epidemiological studies, preclinical rodent studies regularly employ chronic psychosocial stress paradigms to study mechanistic details in mammary carcinogenesis and metastasis. We aimed to review both epidemiological and preclinical studies of psychosocial stress in BC development, focusing on whether chronic psychosocial stress might be a particularly relevant stressor domain.

**Methods:**

For *in vivo* preclinical rodent studies, we searched PubMed for individual studies from 1985 to December 2025 written in English that evaluated effects of chronic psychosocial stress on mammary tumorigenesis and metastasis. We restricted focus to studies that used chronic psychosocial stress protocols. For epidemiological research into psychosocial stress in BC, due to the abundant literature that spans several decades, we searched Medline (PubMed) for English-language systematic reviews and meta-analyses published in the last three decades (1995 to December 2025) in which participants were inquired about exposure to psychosocial stress.

**Results:**

Systematic literature searches identified 29 rodent studies that randomly allocated rodents to chronic psychosocial stress or safety. Twenty-five of the 29 studies found that chronic psychosocial stress increased mammary tumorigenesis and/or metastasis, with the remaining studies reporting more complex effects that reflected variations in study design elements. In contrast, most original research studies included in 14 systematic reviews and meta-analyses of epidemiological research focused on short-lasting, adverse life events, with no consistent associations detected with BC. Epidemiological studies have only partly and unsystematically addressed chronic psychosocial stress.

**Conclusions:**

Findings from preclinical rodent studies indicate that chronic psychosocial stress may be a particularly relevant psychosocial exposure domain in BC development. In contrast, the epidemiological studies we reviewed focused predominantly on acute adverse life events, with limited attention to chronic psychosocial stress. We suggest that this evidence highlights the need to prioritize chronic psychosocial stress in future epidemiological studies of BC etiology. Actionable method components may include a life-course perspective for stress exposure, a multi-level social interaction perspective, inclusion of both chronic and acute stress and their interactions, and inclusion of individuals’ subjective appraisals of distress.

## Introduction

Severe psychosocial stress has long been investigated as a possible antecedent to breast cancer (BC), with theoretical roots dating back to ancient philosophers and empirical studies of associations since the late 19^th^ century (1). Severe psychosocial stress can be conceptualized as the perception of psychosocial environmental demands that disrupt homeostasis and may tax or surpass an individual’s capacity to cope and adapt, if not adequately countered by physiological and behavioral responses (2). After decades of studies where participants have been inquired about their exposure to psychosocial stress, no agreement has been reached in epidemiology regarding its role in BC etiology. Due to small and inconsistent associations, some researchers conclude that women can be reassured that the (often unavoidable) stressors in their lives would not increase their risk of BC (3). In contrast, experimental preclinical studies of BC regularly use psychosocial stress paradigms, most often including chronic stressor setups, to study mechanistic details of BC development and metastasis. Consistent with this, some epidemiological researchers point to preclinical studies to suggest that chronic psychosocial stress also should be studied in human BC development (4–8).

*In vitro* and *in vivo* animal studies and mechanistic studies in individuals with BC indicate that psychosocial stress may be particularly pertinent to study in hormone-dependent BC (estrogen and progesterone), which encompasses 70%-90% of all cases (9). The estrogen system has been found to interact with the hypothalamus-pituitary-adrenal (HPA) and Sympathetic-adrenal-medullary (SAM) stress systems to increase BC risk. When acting on breast tissue, high levels of circulating estrogens over time may contribute to DNA damage and increased cell proliferation and metastasis via formation of estrogen-DNA adducts, reactive oxygen species resulting in oxidative stress, and stimulation of estrogen receptor alpha (ERα) (10). Biological responses to stressor exposure may act in concert with this and interact with estrogen activity to increase the risk of tumor development, including elevated levels of stress hormones such as glucocorticoids and noradrenaline, stimulation of β-adrenergic signaling, translocation of the glucocorticoid receptor (GR) to the nucleus and activation of GR target genes, as well as inflammatory effects upon reactive oxygen species and oxidative stress (10–12). While the mechanisms of these interactions are complex, biological stress responses appear to affect the tumor microenvironment depending on the state of estrogen activity (13). The combination of prolonged estrogen and stress hormone activities, including associated receptor activities, may have synergistic effects upon DNA damage, attenuated DNA repair mechanisms, and enhanced escape from apoptosis, resulting in an increased risk of malignant cell proliferation, carcinogenesis, and metastasis. However, other findings suggest that stress system activation may protect against further BC development and metastasis. Most notably, in estrogen-positive (ER+) BC, enhanced glucocorticoid activity following systemic corticosteroid administration (usually Dexamethasone) leads to increased expression of the GR, which in turn may antagonize ERα activity and decrease tumor proliferation and metastasis (14, 15). That enhanced glucocorticoid activity may inhibit rather than stimulate metastasis indicates the complexity of deciphering the role of psychosocial stress in BC etiology.

This review investigates the impact of chronic psychosocial stress on BC development, focusing on two complementary fields, experimental preclinical research and epidemiology. First, we review original preclinical studies regarding the role of chronic psychosocial stress in mammary tumorigenesis and metastasis. Second, we conduct an umbrella review of systematic reviews and meta-analyses of epidemiological studies assessing participants’ exposure to psychosocial stress, aimed at evaluating the extent to which the field has investigated chronic psychosocial stress in human BC development. Concluding from these reviews that chronic psychosocial stress likely plays a role, we discuss key methodological and theoretical challenges in investigating this exposure domain within epidemiological studies of human BC etiology.

## Methods

In studies of psychosocial stress in humans, chronic stress is typically defined as demanding and distressing experiences that persist over an extended period, often 6 months or more, in contrast to acute stress, where exposure is generally considered to last less than a few weeks, depending on the continuity or frequency of exposure (16). Chronic psychosocial stress includes living in poor or dangerous neighborhood environments, enduring financial strain, enduring interpersonal difficulties such as unfulfilling, conflict-ridden or abusive romantic relationships, enduring adverse childhood experiences in upbringing, repeated sexual and physical abuse and aggression in adulthood, demanding caregiving burdens over time, social isolation, discrimination, and continuing severe health problems (17). In rodent studies, chronic psychosocial stress typically is operationalized as continuous stressor exposure (e.g. social isolation) or daily stress exposure for a few hours each day (e.g. a predator) across two or more consecutive weeks, but with exposure length varying depending on the chronic stressor regime (18).

For *in vivo* preclinical rodent studies, we searched PubMed for individual studies on chronic psychosocial stress in mammary tumorigenesis and metastasis. We restricted our focus to English-language papers published over the last 4 decades (1985 to December 2025) that utilized chronic stressor protocols of a psychosocial nature - specifically, stressors related to the presence or absence of conspecifics or to changing physical environments. We excluded studies using single physical stressors (such as restraint stress alone) (19), but included studies that combined various non-psychological stressors in an unpredictable manner (chronic unpredictable stress), even if they lacked a direct social-relational component, assuming these stressor regimes may represent the stress of frequently changing environments in humans. We only selected studies that (i) were reported in detail in a scientific article, excluding those reported only as an abstract, (ii) focused on mammary tumors/BC cells and their growth and metastasis, excluding other tumor types or outcome measures, (iii) used chronic stressor exposure protocols lasting at least 10 days, and (iv) included female rodents, as human BC is strongly associated with females. Regarding studies that used both male and female rodents, we reported findings only for the females. To search for rodent studies, we used the following MeSH search terms and Boolean algebra commands: (rodent* OR mice OR mouse OR rat OR preclinical OR xenograft OR “orthotopic model” OR “animal model” OR “murine model” OR “*in vivo*”) AND (“breast cancer” OR “mammary carcinoma” OR “breast tumor” OR “mammary gland cancer” OR “breast adenocarcinoma” OR “mammary neoplasm”) AND (“chronic stress” OR “chronic unpredictable stress” OR “chronic unpredictable mild stress” OR “social isolation” OR “individual housing” OR “maternal separation”).

For epidemiological research into psychosocial stress in BC, giving the extensive literature spanning several decades, we searched Medline (PubMed) for English-language systematic reviews and meta-analyses. We restricted the inclusion of papers to those published in the last three decades (1995 to December 2025), because reviews prior to 1995 tended to have a narrative rather than meta-analytic character. We required systematic reviews and meta-analyses to use a systematic search approach and methodological quality criteria to select eligible original studies, and we excluded reviews where authors selected original articles based on other criteria (e.g. subjective selection of what they considered the most “relevant” studies) [e.g (4, 20)]. Included were systematic reviews and meta-analyses that focused on external stressors in terms of events, sequences of events, conditions, or changes in the psychosocial environment, as reported by study participants upon inquiry. We placed no restrictions on which environmental situations, events, conditions, or changes to focus on, provided they were external or embedded in nature; we therefore excluded internal psychological stressors such as anxiety, depression, and personality style. We excluded night shift work, because the rationale for studying this in BC relates more to biological processes (chronobiological disturbances/disruptions of biological rhythms) than to psychosocial stress (21). We summarize the overall approach, design, focus, and findings of identified systematic reviews and meta-analysis. We also describe the types of psychosocial variables and measurement approaches used, the duration of stressor exposures, the instruments used, and associations with BC risk. For the years since the last review/meta-analysis was published (identified to 2019), we searched PubMed and PsycINFO for subsequently published original epidemiological studies on psychosocial stress. In searching for epidemiological systematic reviews and meta-analyses, we used the following MeSH search terms and Boolean algebra commands: (“psychological stress” OR “psychosocial stress” OR “life events” OR “chronic stress” OR “sexual abuse” OR “sexual assault” OR “physical abuse” OR “childhood abuse” OR “adverse childhood experiences” OR “intimate partner violence” OR rape OR racism OR discrimination OR “work stress” OR “job strain” OR “financial strain” OR “financial difficulties” OR “social isolation” OR “social support”) AND (“breast cancer”) AND (systematic review” OR “meta-analysis”).

In both thematic searches, we screened references in identified papers for additional relevant articles.

## Results

### Experimental studies in rodents

**Figure 1:**
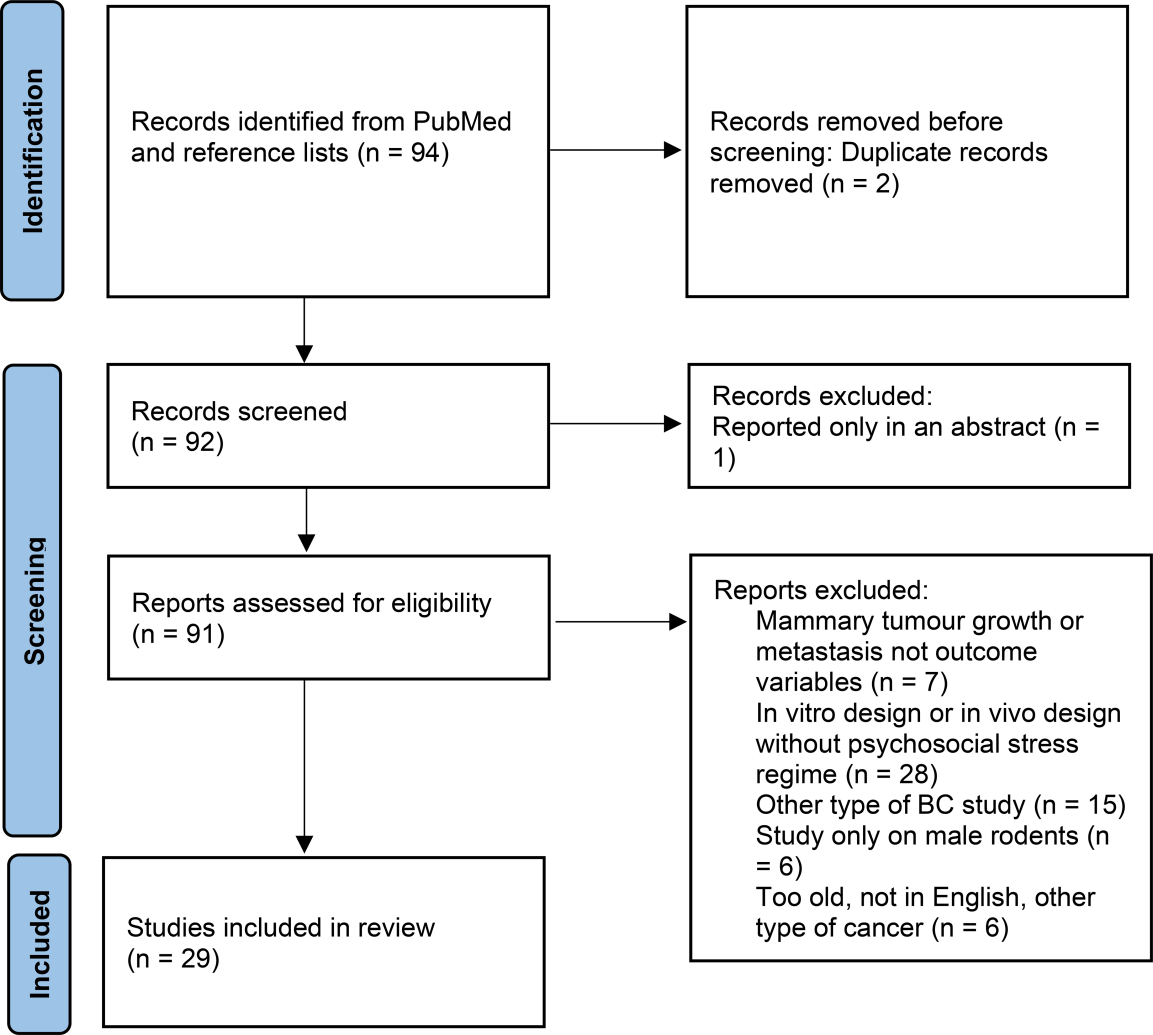
PRISMA flow diagram for experimental, preclinical studies on chronic psychosocial stress in breast cancer development in female rodent models.

We identified 29 original studies on chronic psychosocial stress in BC etiology in female rodents, published from 1989 to 2025 (**Figure 1**, **Table 1**). The main stressor protocols used in these studies were social isolation (14 studies), various operationalizations of chronic unpredictable mild stress (CUMS) (12 studies), combined social isolation and CUMS (1 study) and maternal separation (2 studies). The studies exposed rodents to stressors for a period ranging from 11 days to a lifetime, used inbred and manipulated (e.g. transgenic, knockout) rodent models, injected specimens with mammary tumor cells, and randomized individuals to chronic stress or control groups.

**Table 1:** Original studies of mammary tumor development and metastasis in female rodent breast cancer models exposed to chronic psychosocial stress.

Authors	Rodent model	Experimental psychosocial stress condition	Findings
Kerr et al. (1999) (24)	Female DD/S mice injected with Shionogi female mammary carcinoma (SC115)	Social isolation + daily novelty stress: mice initially housed in groups were moved to individual housing for 4 weeks (and vice versa in control group), plus – 15 min per day moved to novel housing condition (acute novelty stressor)	(i) Significantly increased tumor growth rate and tumor weight for isolation, but (ii) decreased tumor growth rate and tumor weight in mice also exposed to both isolation and daily acute stress
Hermes et al. (2009) (34)	Female Sprague–Dawley/Norwegian rats, natural rats with no injections/manipulations	Life-long social isolation (vs group-housing)	Significantly (3.3 times) increased risk for developing at least one malignant tumor, increased tumor growth, 84 times increased tumor burden (weight of all solid tumors)
Williams et al. (2009) (35)	Female C3 (1)/SV40 T-antigen transgenic mice	Social isolation vs group-housing from weaning until 21 weeks of age	Significantly increased tumor incidence and tumor size in socially isolated animals
Boyd et al. (2010) (36)	Female BALB/c Mice injected with DMBA carcinogens in adulthood	Maternal separation in neonatal period for 4 hours/day for the first 3 weeks of life	Significantly enhanced mammary tumorigenesis and invasive mammary carcinomas
Hasen et al. (2010) (25)	Female wild type mice and female mice with deleterious mutations in the P53 risk gene	Social isolation (individual housing) for 11 weeks that included period for mammary gland development in puberty	Reduced early mammary development, reduced survival at 1 year of age, and reduced incidence of developing mammary tumors
Madden et al. (2013) (37)	Female severe combined immunodeficiency mice injected with MB-231 breast cancer tumor cells	Social isolation for 5 weeks	Significant but transient increase in tumor growth
Volden et al. (2014) (38)	Female C3(1)/SV40 T-antigen FVB/N (TAg) mouse model of triple-negative breast cancer	Social isolation for 18 weeks	Significantly larger tumor burden
Qin et al. (2015) (39)	Female BALB/c mice injected with 4T1 breast cancer cells	Social isolation (individual housing) for 3 weeks	Significantly faster tumor growth and 5 times increased tumor volume
De la Roca Chiapas et al. (2016) (40)	Female Sprague Dawley rats injected with the mammary carcinogen N-methyl-N-nitrosourea	Social isolation (individual housing) for 10 weeks	Significantly increased tumor incidence and tumor weight
Sumis et al. (2016) (41)	Female C57BL/6 mice, Atg7^+/−^ and wild type mice injected with DMBA breast cancer cells	Social isolation (individual housing) for 14 weeks	Significantly increased tumor frequency and shorter time to tumor development, and nominally increased tumor burden
Lu et al. (2017) (42)	Female BALB/c mice injected with 4T1 breast cancer cells	Chronic unpredictable mild stress for 4 weeks: Thermal stimulation, swimming in cold water, fasting, water deprivation, day-night reversal, horizontal oscillation, catching the tail of the mouse, and hanging up.	Significantly increased tumor volume and tumor growth speed
Budiu et al. (2017) (22)	Female BALB/c Immunocompetent mice injected with 4T1 mammary cancer cells	Social isolation for 4 weeks or until tumor size reached 100 mm^3^	Increased death rate indicating more aggressive tumors. No difference in mammary tumor weights, significant increase in endothelial marker CD31 (a measure of tumor angiogenesis) in primary tumors and in lung metastasis.
Chen et al. (2018) (43)	Female Balb/C mice, C57BL/6j mice, NOD scid gamma mice, and transgenic MMTV-PyMT mice injected with human breast cancer cell lines MCF-7 and MCF-7/GFP	Chronic unpredictable stressors for 11 days: cage tilt, isolation, crowding, rapid light–dark changes, damp bedding, and overnight illumination	Significant promotion of lung metastatic colonization of circulating breast cancer cells
Zhou et al. (2020) (44)	Female BALB/c mice injected with 4T1 breast cancer cells	Social isolation for 4 weeks	Significantly increased tumor growth and weight and lung metastatic nodules
Dawes et al. (2020) (23)	Female mice expressing the mouse mammary tumor virus polyoma top-T antigen	Social isolation for 8 weeks plus restraint stress 2hr per day for three days in two of these weeks (acute stressor – administered to reduce habituation to isolation in this mouse strain)	The double stressor exposure reduced mammary tumor burden in association with increased tumor cleaved caspase-3 expression, indicative of increased cell apoptosis
Garcia-Laguna et al. (2021) (5)	Female Wistar rats induced with DMBA cancer cell lines	Maternal separation 6 hours/day for 20 days	Significantly increased incidence of preneoplastic changes and breast carcinogenesis
An et al. (2021) (45)	Female BALB/c mice injected with 4T1 breast cancer cells	Chronic unpredictable mild stress for 4 weeks: sleep deprivation, cage shaking, damp bedding, isolation or crowding, and overnight illumination	Significant effects: faster tumor growth, increased and more severe metastasis of breast cancer cells in the lungs
Berry et al. (2021) (46)	Female MMTVNeuTg mice, a mouse model of breast cancer susceptibility that overexpresses the ErbB2 receptor	Social isolation for 20 weeks	Significantly increased number of breast lumps
Ye et al. (2023) (47)	Female Balb/c and C57BL/6 mice with injection of breast cancer cell lines 4T1 and E0771	Chronic unpredictable stress for 2 weeks: tail pinch, inescapable foot shocks, wet bedding with cage tilt, placement in a 4 °C cold room, noise in the room for, stroboscopic light, restraint, cage rotation for, light on overnight, and light off during the day	Significantly increased tumor weight, tumor volume, and lung metastasis
Pan et al. (2023) (28)	Wild-type BALB/c female mice and PyMT-MMTV transgenic mice injected with 4T1 breast cancer cell line	Chronic unpredictable mild stress 6 hours per day for 3 weeks: cage tilt, wet padding, padding from aggressor and more.	Profoundly (x 3 times) increased tumor volume and significantly more severe lung metastasis of breast cancer cells
Andrade et al. (2023) (48)	Female Sprague Dawley rats injected with DMBA mammary tumors	Social isolation for 9+ weeks.	Significantly increased local mammary tumor recurrence
Chen et al. (2023) (49)	Female BALB/c mice injected with 4T1 breast cancer cells	Social isolation plus chronic unpredictable mild stress (cage tilt, moist cage, sawdust bedding, physical restraint, foreign object exposure, water deprivation, light/dark perversion, tail pinch, overnight illumination, overhang, white noise) for 12 weeks	Significantly increased tumor size (x 2) and tumor weight
He et al. (2024) (50)	Female BL/6 mice injected with PyMT breast cancer cells	Chronic unpredictable mild stress for 3 weeks: tail pinch, physical restraint; cold swimming, noise stress, food deprivation (overnight); water deprivation (overnight); moist bedding; removal of all bedding, cage tilt, stroboscopic lights (overnight)	Significant increase (doubling) of lung metastasis
Lin et al. (2024) (51)	Female BALB/c mice injected with 4T1 breast cancer cells	Chronic unpredictable mild stress (24-hour illumination or darkness, cold/thermal/noise stimulation, red light, shaking, no padding, no water/food/padding) and chronic restraint stress for 4 weeks	Significantly increased tumor growth and metastasis
Liu et al. (2024) (52)	Female xenograft (immunodeficient) mice injected with triple negative breast cancer (TNBC) cells	Chronic unpredictable mild stress (social isolation, cage tilt, overnight illumination, rapid light-dark changes, damp bedding) for 5 weeks	Significantly accelerated proliferation, migration and invasion of TNBC cells, and enhanced tumor growth and lung metastasis
Qin et al. (2025) (47)	Female BALB/c mice and male C57BL/6 mice injected with luciferase gene-tagged 4T1 murine breast cancer cell	Chronic unpredictable mild stress for 8 weeks: day-night reversal, cold-water swimming, crowd-feeding, water and food deprivation, an empty water bottle, a 45°cage tilt, a tail clamp, a self-made plastic seal tube, and a wet pad	Significantly accelerated tumor tumorigenesis and metastasis, including a doubling of overall tumor weight
Liu et al. (2025) (53)	BALB/C nude female mice with injection of MDA-MB-231 triple negative breast cancer cells	Chronic unpredictable stress for 8 weeks: Wet bedding, light overnight, dark/light treatment on nights/days, forced swimming, deprivation of food and water, social isolation, cage tilting, restraint stress	Significant escalation in tumor size (50% larger) and tumor weight (5 times higher)
Dees et al. (2025) (54)	Female BALB/c mice injected with EMT6 breast cancer cells	Chronic unpredictable stress for 24 days, variations between morning stressors for 1 hr (shaking of the cage, predator call, or confinement to a 50 mL tube) and overnight stress (45°C tilted cage, wet bedding, or simulated 24-h daylight)	8 times increased tumor volume
Wang et al. (2025) (55)	Female BALB/c mice injected with 4T1 breast cancer cells	Chronic unpredictable mild stress for 4 weeks (details of stressor regime not stated)	Significantly increased metastasis to the lungs

In 25 of 29 studies, animals exposed to chronic stress demonstrated adverse tumor development and progression, including increased tumor growth (size and weight) and/or increased colonization and metastasis (**Table 1**). These associations were consistent across all chronic stressor protocols, various BC animal models, and different timing of exposure (early life, adolescence, adulthood, lifetime). The assumption that carcinogenic effects were promoted by chronic psychosocial stress was supported by findings of concomitant alterations in numerous components of the SAM system, HPA axis, and immune system, occurring prior to tumor growth and metastasis.

A difference was evident between studies employing CUMS and social isolation as stressor protocols. Considering the subset of these studies that reported unequivocal carcinogenic effects, the authors reported increased tumor size, weight, or growth in 9 out of 12 CUMS studies and 12 out of 14 social isolation studies. However, metastasis was reported in 9 out of 12 CUMS studies but only in 1 out of 14 social isolation studies, suggesting that compared to social isolation, CUMS may be more conducive to metastasis.

Four rodent studies reported more nuanced findings than an unequivocal acceleration of tumor growth and/or metastasis following exposure to chronic psychosocial stress. Budiu et al. (22) found no differences in primary mammary tumor weights or metastasis in female BALB/c immunocompetent mice following social isolation. However, they observed increased mortality in the socially isolated mice and increased expression of the endothelial cell adhesion molecule CD31, which indicated increased angiogenesis and rapid tumor growth. The authors concluded that primary tumors were more aggressive in their socially isolated mouse model. Dawes et al. (23) combined social isolation with intermittent acute stressor exposure (restraint stress) and observed reduced mammary tumor burden in association with increased tumor cleaved caspase-3 expression, indicating increased cell apoptosis. The authors suggested that any stress-induced increase in metastatic lesions may have been countered by a reduction in tumor growth and the associated activation of tumor inhibitor pathways by intermittent (acute) novelty exposure. Kerr et al. (24) reported that social isolation significantly increased tumor growth rate and weight in female mice, but decreased tumor growth when social isolation was combined with acute daily stress. Hasen et al. (25) reported that mice exposed to individual housing for 11 weeks exhibited reduced mammary tumorigenesis compared to non-stressed mice. They commented that their chronic stress paradigm was initiated at puberty, which inhibited mammary development itself and reduced survival. The authors reasoned that the timing of the stressor exposure at puberty represented a confounding factor contributing to their finding of reduced mammary tumorigenesis.

**Figure 2:**
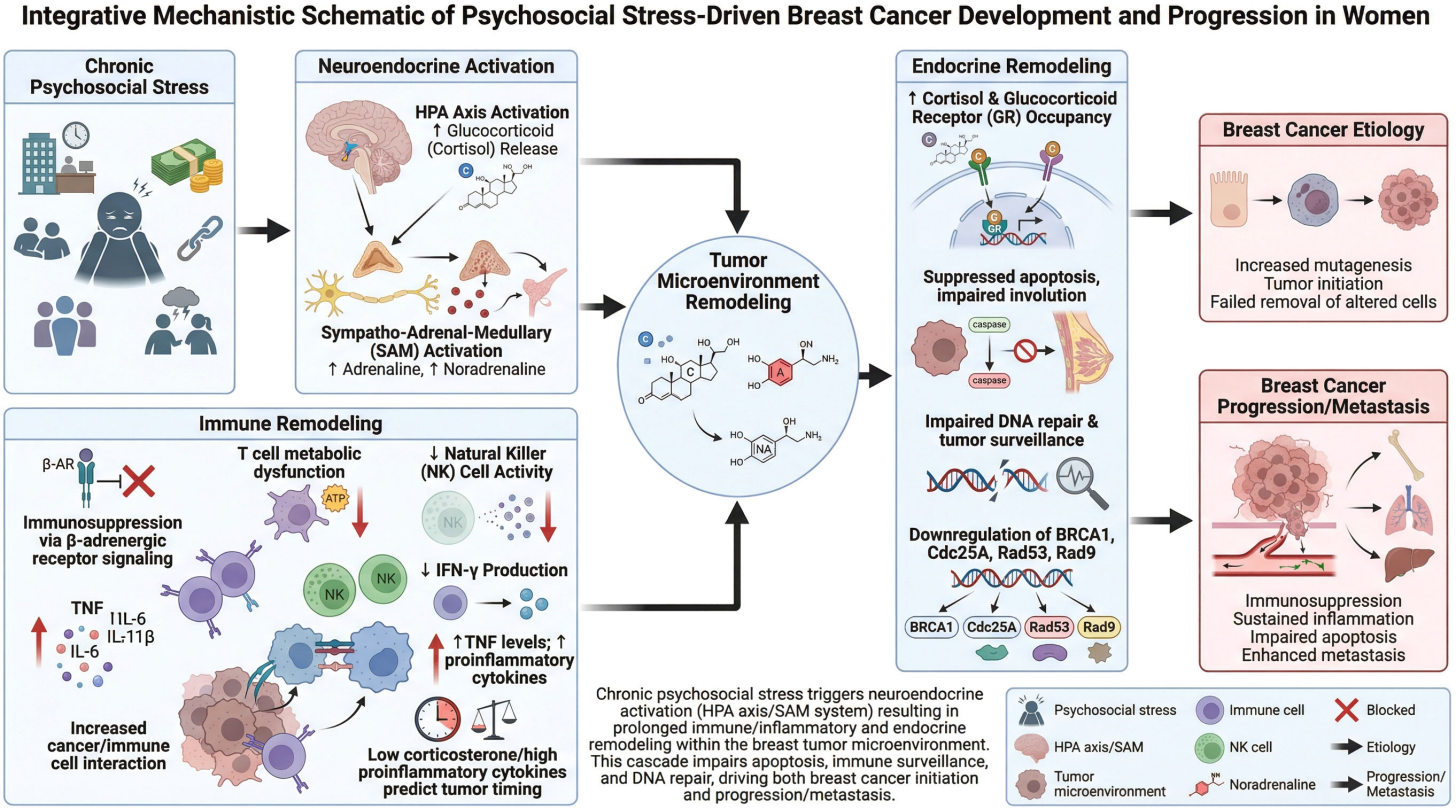
Integrative mechanistic schematic model.

A common theme across rodent studies using chronic stress paradigms is the investigation of mechanistic details of BC progression, specifically those involved in apoptosis modulation, altered regulation of immune function, and changes in DNA repair mechanisms (4). These are considered downstream effects of stressor-associated activation of main stress-processing systems, most notably elevated HPA axis engagement - with increased glucocorticoid release - and heightened sympathetic nervous system activity with elevated adrenaline and noradrenaline release. Over time, these activations negatively affect immune system processes in the tumor microenvironment, including immunosuppression following stress-induced *β*-adrenergic receptor signaling (which may serve as a checkpoint for immune responses), T cell metabolic dysfunction, a decline in natural killer cell activity, decreased IFN-gamma production, and increased inflammatory activity characterized by higher TNF-levels and increased interaction between cancerous and inflammatory immune cells (26, 27). Additionally, low corticosterone and high proinflammatory cytokine levels predict the timing of tumor development (28–30). Other changes mediating the effects of chronic stress on BC progression in rodent models include increased cortisol and GR occupancy, which may suppress the removal of genetically altered cells through apoptosis and involution (growth-reversal) (4). An endpoint for increased BC propensity following chronic stress appears to be impaired tumor surveillance and DNA repair capabilities that cause or fail to inhibit DNA mutagenesis (31, 32), at least partly reflecting cortisol- or GR--contingent downregulation of genes including *BRCA1, Cdc25A, Rad53* and *Rad9* (33). These general mechanisms are illustrated in **Figure 2**.

### Epidemiological studies of psychosocial stress in breast cancer

**Figure 3:**
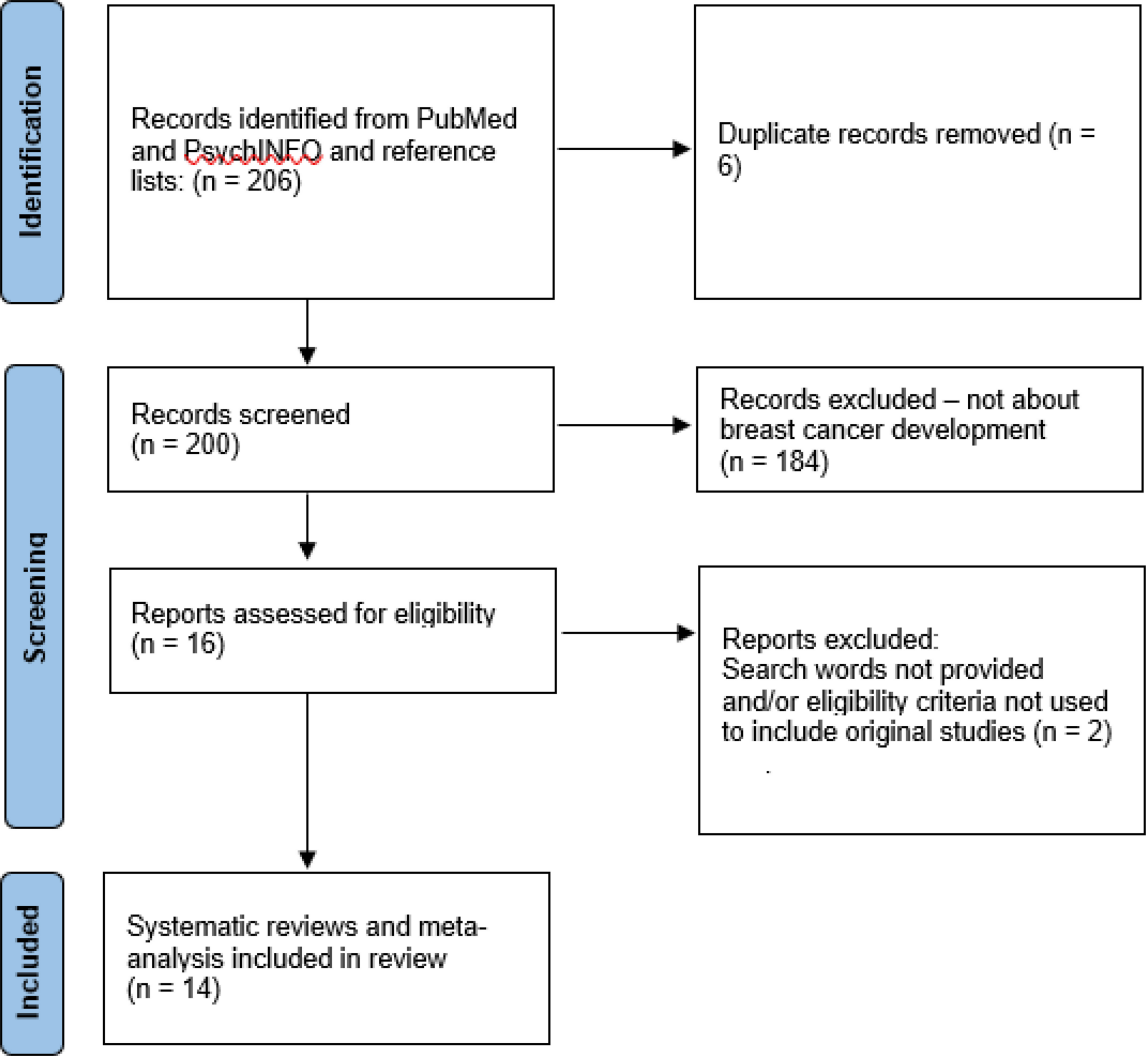
PRISMA flow diagram of systematic reviews and meta-analyses of exposure to psychosocial stress in human breast cancer development.

The literature search for epidemiological studies of psychosocial stress as risk factors for BC identified 14 systematic reviews and meta-analyses (**Figure 3**, **Table 2**), published between 1999 and 2019. Eleven reviews/meta-analyses had a predominant focus on adverse life events, two focused on job strain and one on adverse childhood experiences. The 14 reviews together included 103 unique studies (duplicates excluded). Of these, 53 were retrospective case-control studies or limited prospective studies with retrospective data acquisition, 41 were prospective longitudinal studies, and 9 were registry linkage studies. A subset of the retrospective studies inquired about stressful experiences within a circumscribed period several decades earlier (childhood, second world war). The remaining bulk of retrospective studies acquired data for specified extensions backwards in time from the assessment date, varying from 1 month to 15 years (median 3.5 years, mean 4.6, SD = 3.7). Follow up periods in prospective studies varied from 4 to 40 years (median 11 years, mean 12.3, SD = 7.7).

**Table 2:** Systematic reviews and meta-analyses of associations between psychosocial stress and human breast cancer development.

Authors	Studies, design, participants (n)	Psychosocial stressor measurement	Findings of associations with breast cancer
McKenna et al. (1999) (63)	12 retrospective case-control studies. Studies included: 1970-1996 Total n=4,382	Stressful life events measures: Life Experiences Survey, Life Events Inventory, the Holmes-Rahe Social Readjustment Rating Scale (SRRS), the Holmes-Rahe Schedule of Recent Experience, the Life Events and Difficulty Schedule (LEDS), and investigator-constructed checklists^2^ Exposure period retrospective studies: 1–5 years pre-diagnosis (n=8), 6–10 years (n=1), 11–20 years (n=1), and unknown (n=2).	Stressful life events: Hedges g (effect size in SD) = 0.25, 95%CI 0.08.0.42) (small effect)
Petticrew et al. (1999) (59)	29 studies: 14 retrospective case–control, 14 limited prospective studies, 1 prospective study Studies included: 1966-1997 Total n=14,200	11 studies of bereavement (yes/no) 15 studies of other life events. Exposure periods varied from 1 year to entire life prior to diagnosis/measurement Measures: Widowhood/divorce (yes/no) (n=10), LEDS (n=1), Holmes and Rahe Social Readjustment Rating Scale (SRRS) (n=5), Life event inventory (n=2), Schedule of recent experiences (SRE) (n=3), non-standardized or psychiatric interviews (n=3), Life event scale (n=1), not stated/other (n=6) Exposure periods: 16 studies 1–5 years pre-diagnosis, 7 studies 6–10 years, and 4 studies lifetime.	Overall sample: No associations for bereavement. Significant association for other life events – OR = 2.63, 95%CI 2.34-2.96. Considering only high-quality studies: No associations for either bereavement (OR = 0.9 ns) or other life events (OR = 0.8 ns).
Butow et al. (2000) (3)	16 studies: 2 record linkage studies, 7 limited prospective studies, 7 case-control studies Studies included: 1975-1996 Total n=59,387	Stressful life events. Measures: Widowhood/divorce (yes/no) (n=2), LEDS (n=2), Holmes and Rahe Social Readjustment Rating Scale (SRRS) (n=4), Life event inventory (n=5), Schedule of recent experiences (SRE) (n=1), non-standardized or psychiatric interviews (n=3). Exposure periods retrospective and limited prospective studies: 13 studies 1–5 years pre-diagnosis, 3 studies 6–10 years, and 1 study lifetime	(a) Widowhood/divorce negatively associated in one study – OR = 0.83, 95%CI 0.75.0.92, but no associations in a second study. (b) Two studies using LEDS: (i) positive correlation between the most severe life events and breast cancer in one study (r=0.28), (ii) positive associations for severely threatening life events in second study (adjusted OR = 15.00, 95%CI 3.74–60.44) (c) Three limited prospective studies using SRRS or SRE: two found no associations, the third found a significant negative association with life event scores. (d) Of 7 case-control studies, a positive association was found in 3 and no association or results were unclassifiable in 4.
Duijts et al. (2003) (57)	27 studies: 10 retrospective case–control, 4 prospective case–control, 9 limited prospective cohort, 4 prospective cohort. Studies included: 1966-2002 Total n=7,666	Stressful life events. Measures: Yes/no questions – death of parents/widowhood/war experiences (n=2 studies), LEDS^1^ (n=5), Life event inventory (n=2), Self readjustment rating scale (n=1), record linkage (n=3), Self-made interviews/questionnaires (n=7), job strain – Job content questionnaire (n=1), Holmes and Rahe Social Readjustment Rating Scale (SRRS) (n=1), Other/not stated (n=5) Exposure period: 1–5 years pre-diagnosis (n=13), 6–10 years (n=4), 20–40 years (n=2), and lifetime (n=1), and unknown (n=7).	Positive significant (p < 0.05) associations for: overall stressful life events (OR = 1.77, 95% CI 1.31–2.40), death of spouse (OR = 1.37, 95% CI 1.10 –1.71) and death of relative or friend (OR = 1.35, 95% CI 1.09 –1.68). No associations for health difficulties (own and others’), divorce, financial status, or change in environmental status.
Nielsen & Grønbæk (2006) (56)	13 studies with prospective design: 10 cohort and 3 case-control. Studies included: until 2005 Total n=338,630	Life events, work stress, perceived stress. Yes/no responses: Divorce/loss of husband, parent dead in childhood, cancer in a child, death of child, death of son in war/accident (n=7). Work stress - Job content questionnaire (n=1). Perceived stress - various measures (n=4). Stressful life events – modified Holmes and Rahe Social Readjustment Rating Scale (SRRS) (n=1). Exposure periods: Follow up periods: 8–24 years (3 studies not stated)	8 studies found no significant associations, including on work stress. Among the 5 other studies with significant findings, positive associations were reported for death of mother in childhood (HR 2.13, 1.02–4.55), increase in major life events (HR 1.35, 1.09–1.67), and perceived stress last 5 years (HR 2.0, 1.1–3.5), one-event increase in major life stressors (HR 1.35, 1.09–1.67), while 2 studies found negative associations (reduced risk) for high vs low perceived stress (OR = 0.60, 0.37–0.97), and loss of husband by death (OR = 0.8, 0,7.1.0).
Chida et al. (2008) (62)	31 prospective studies and registry studies. Studies included: 1966-2007 Total n = not stated	Psychosocial stress: several categories were identified and collapsed: External stressors (stressful life events, severe chronic stress, daily stress and poor social support, n = 15); and “internal stressors” such as emotional distress; stress prone personality and coping style (n=16). Data presented did not include specific estimates for exposure stressors. Follow up periods in prospective studies of (external) stress exposure: 1–5 years (n=3), 1–10 years (n=8), 18–21 years (n=2).	No association between psychosocial stress and breast cancer development in meta-analysis (HR = 0.99, 95%CI 0.92-1.06).
Santos et al. (2009) (58)	Eight studies: 6 retrospective case-controls and 2 prospective cohorts. Studies included: 1982-2007 Total n = 66,612.	Divorce/widowhood (yes/no) (n=3) Stressful life events: LEDS (n=2), Holmes and Rahe Social Readjustment Rating Scale (SRRS) (n=2), self-made questions of subjective stress (n=1) Exposure periods: 5 years (n=5), 10 years (n=1), 15 years (n=1), lifetime (n=1). Follow up period in cohort studies: 6 and 25 years	No significant (p<0.05) associations for widowhood, divorce or Intensity/frequency of stressful life events.
Lin et al. (2013) (67)	7 studies: 4 case–control and 3 prospective cohort. Studies included: 1995-2012 Total n=99,807	Striking life events. Measures: Holmes and Rahe Social Readjustment Rating Scale (SRRS) (n=3), LEDS (n=2), self-made questions (n=2). Exposure periods: 1–5 years (n=7) Follow up periods in prospective studies: 5, 7.6 and 15 years	Meta-analysis: Significant association with striking life events – OR = 1.51, 95% CI 1.15 - 1.97, P = 0.003, and with severely striking life events OR 2.07, 95%CI 1.06–4.03).
Heikkila et al. (2013) (64)	12 prospective cohort studies from the same European research consortium. Studies included: 1985-2008 Total n = 123,300.	Job strain/Work stress Measures: Job Content Questionnaire (JCQ) and Demand-Control Questionnaire (DCQ). Follow up period prospective studies: Mean of 12 years	Work stress, high job strain was not associated with risk of breast cancer, HR = 0.97, 95%CI 0.82 to 1.14.
Holman et al. (2016) (65)	9 studies: 5 prospective cohort and 4 retrospective case control. Studies included: 1998-2015 Total n = 119,100.	Adverse childhood experiences (ACE). Measures: Various instruments and interview approaches to measure an extended range of ACEs: sexual, physical and psychological abuse, domestic violence, parental mental illness, substance abuse, divorce and death, prolonged financial difficulties, serious conflicts, incarceration, and summary ACE scores. Exposure period: Childhood (up to 69 years earlier)	Eight of 9 studies investigated cancer in general as outcomes, just 1 study specifically reported for breast cancer. ACEs were associated with “any cancer type” in adulthood, but ACEs not associated with breast cancer as specific outcome (investigated in 1 study only).
Chiriac et al. (2018) (61)	40 studies^5^: 5 retrospective, 15 limited prospective and 10 prospective. Studies included: 1996-2016 Total n > 650,000	Life events. Measures: Holmes and Rahe Social Readjustment Rating Scale (SRRS) (n=6), LEDS (n=4), Life events inventory (n=4), self-made questions (n=2), Life event questionnaire (n=1), List of Threatening Experiences (n=1), Reeder Stress Inventory (n=1), Bahnson psychosocial risk profile (n=1), yes/no questions (n=8), self-made questions/interview (n=14). Exposure periods retrospective studies (including limited prospective: 1 month (n=1), 1–5 years pre-diagnosis (n=16), 6–10 years (n=7), 11–20 years (n=2), > 40 years to lifetime (n=2), unknown (n=2). Follow-up period prospective studies: 8–10 years (n=2), 11–20 years (n=2), 21–30 years (n=3), 31–50 years (n=2), unknown (n=1)	Modest association with one or more adverse life events in 22 studies, no associations in 11 studies, and negative associations in 6 studies. Herein: Prospective studies - 4 studies with positive, 2 with negative and 4 with no associations. Limited prospective studies - 8 studies with positive, 3 with negative and 4 with no associations. Retrospective studies - 10 studies with positive, 1 with negative and 3 with no associations. Findings of positive associations were for: Number of negative events (n=3 studies), emotional losses, major life events, Holocaust, personal illness, legal problems, death of relative/friend (n=7), divorce, chronic stress, stress in family, high perceived stress, highly threatening stress, severe losses, severe deficits in childhood. Findings of negative associations for: Divorce, high stress, number of life events (n=2), death of relatives, contextual threat, severe losses.
Bahri et al. (2019) (60)	11 prospective cohort studies. Studies included: until 2018 Total n=864,619	Life events. Measures: Stressful life events - LEDS (n=2), Life events inventory, (n=1) self-made questions (n=3). Stress of daily activities - self-made questions (n=1). Death of mother or father in childhood or adolescence, death of a child, death of partner - yes/no questions (n=4). Follow up periods in prospective studies: 1–5 years (n=3), 6–10 years (n=2), 11–20 years (n=5), 40 years (n=1).	6 studies found significant associations for: Mother dead in childhood (RR 2.56, 1.59–4.35); death of a child (RR 1.18, 1.01–1.37); stressful life events (RR 1.07, 1.00–1.15, RR 2.1, 1.2-3.7, RR 1.12, 1.01-1.25); parent dead in adulthood (RR 1.1, 1.0-1.3). 5 studies found no associations.
Yang et al. (2019) (66)	9 prospective cohort or case-control studies^6^. Studies included: until 2018 Total n = 290 380	Job strain/Work stress Measures: Established questionnaires for job strain, or interviews. Follow-up period in prospective study specifically on breast cancer: 13 years	A positive association between work stress and cancer in general, RR = 1.17 (95% CI: 1.09–1.25). Only 1 study with specific data on breasts cancer: high vs low job strain positively associated with breast cancer, HR = 1.4; 95%CI 1.1-1.9.
Kruk et al. (2019) (68)	6 studies on breast cancer: 4 retrospective case-control and 2 prospective. Studies included: 2011-2018 Total n = 110 000	Adverse life events (6 studies), child trauma (1 study), chronic stress (2 studies). Exposure periods retrospective studies (including limited prospective: 1–5 years pre-diagnosis (n=4). Follow-up period prospective studies: 7–9 years (n=2)	Life events: Death of family member – 2 positive studies and 2 studies w/no associations. Injury/illness – 1 positive and 2 no associations. Total life event scores: 2 positive a 1 w/no associations. Divorce – 1 study w/no associations Child trauma: 1 study: OR = 1.48 (1.14-1.91) Chronic stress: 1 study w/no associations, 1 study w/association [OR = 2.01 (1.52-2.67)]

Systematic reviews and meta-analyses of psychosocial stress exposure in BC development that provided information on search strategies and that used methodological quality check criteria. ^1^LEDS – Life events and difficulties schedule, used by authors for, respectively, the following last years prior to diagnoses: last 2–5 years. ^2^Authors did not provide information instruments used in each study, or on the time periods for stress exposure that were inquired about when using these instruments. ^3^ns = not significant. ^4^A large control group in a registry study with 141,798 participants not included in total participant number. ^5^12 studies excluded here since they did not focus on stressful external events but instead on personality, social support, and nursing. ^6^The 12 prospective studies on work stress reviewed by Heikilla et al. (64) were considered as one study in this meta-analysis.

Authors’ conclusions from the systematic reviews and meta-analyses were that psychosocial stress is not indicated as an important risk factor for BC incidence (56); stressful, adverse life events – which is the most studied stress exposure category - as a whole are not associated with risk of BC (57–59) or only weekly/possibly associated with it (60, 61); and the evidence for a relationship between psychosocial factors and BC is only modest or weak (3, 62, 63). Authors of three reviews concluded slightly more positively: that work stress as well as adverse childhood experiences may be modestly associated with at least some cancer types, but that this is more unclear for BC (64–66), and that high-intensity stress has a borderline association with the development of BC (67). Authors of one review concluded clearly positively that severe life events play a prominent role in BC (68). Moreover, McKenna (63), p. 520-521] summarized authors’ conclusions from several narrative reviews prior to 1999 (69–73): “*These reviews tend to fall into two categories: (a) those that conclude there is no association and often offer a host of methodological and conceptual weaknesses that may have concealed an association, and (b) those that weakly support a connection*.

### Adverse life events

Eleven of the 14 systematic reviews and meta-analyses had a primary focus on adverse life events, which are characterized by a relatively brief exposure duration (3, 56–63, 67, 68). To measure adverse life events, studies used self-made questions, semi-structured open interviews or simple yes/no questions. Also frequently used were checklists of life events such as the Life event inventory, Schedule of recent experiences, Life event scale, Life event questionnaire, and the Holmes-Rahe Social Readjustment Rating Scale. Typical adverse life events captured with these inquiries were death of a partner/spouse, parent, child, or friend, divorce and widowhood, personal injury or illness, retirement from work, pregnancy and giving birth, change in work, business readjustment, losing a job, and negative changes in financial state. The negative life events that authors most frequently, although far from unanimously, reported to be associated with BC risk were widowhood/divorce and bereavement/death of husband, mother, child or friend. As evident from authors’ own conclusions and the summaries in **Table 2**, no consistent evidence has appeared for a role of adverse life events in BC etiology.

Several individual studies included in the systematic reviews and meta-analyses used the Life Events and Difficulty Schedule (LEDS) (74), a detailed, structured interview approach often considered the gold standard instrument for assessing psychosocial stress. The LEDS is designed to measure both short-lasting life events and ongoing life difficulties. However, the LEDS conceptualizes ongoing difficulties as dependent on - and representing the context for - life events, rather than as a separate domain of psychosocial exposure. Most researchers who have used the LEDS in studies of BC have done so to quantify life events, with little focus on ongoing difficulties. A few exceptions exist, which we return to below when considering epidemiological studies that might have targeted chronic psychosocial stress.

### Job strain

Two meta-analyses focused on work stress or job strain, together including 20 original studies (64, 66) (**Table 2**). Job strain includes exposure to high demands or high work pressure, low job control, or long working hours. Since job strain may develop and persist for a long period (years, decades), it would qualify as chronic stress. One meta-analysis on job strain (64) found no associations with BC, while the other reported, first, a weak association with cancer in general, and second, a moderate positive association with BC in the only study that reported selectively on this cancer type (66, 75).

### Adverse childhood experiences and sexual and physical abuse in adulthood

Holman et al. (65) conducted a systematic review on the association between adverse childhood experiences and the risk of any cancer in adulthood. Based on 12 included studies, the authors reported a significant, positive association between summary scores for adverse childhood experiences and adult cancer risk. The review included only one study evaluating the risk specifically for BC as a function of adverse childhood experiences (76). That study analyzed the association between sexual and physical abuse in childhood, adolescence, and adult life (yes vs no for each period) with BC, and found associations only for exposure in adulthood, conveying 18%-24% increased BC risk, but no associations for childhood or adolescent exposures. A second study on adverse childhood experiences was included in a systematic review by Kruk et al. (68). In their case-control study of 491 patients with BC and 512 controls, they reported that a broad spectrum of adverse childhood experiences (dysfunctional family, adult responsibilities in childhood, parental divorce, loss of a parent, serious health problems, negligence, abuse, and violence) increased the risk of BC by 48% in patients compared to controls. Moreover, Stein and Barret-Connor (77) reported elevated BC risk in older people with a lifetime history of sexual abuse or assaults (OR = 2.1, 95%CI 1.12-4.33). A dose response association also was indicated, with women exposed to multiple episodes of sexual abuse/assault having close to three times increased risk of BC compared to those without such exposures. Within the exposed sample, 59% first experienced sexual abuse before they turned 18.

### Caregiving

In a single study, Kroenke et al. (78) investigated informal adult caregiving for family members – a possible proxy for chronic psychosocial stress. The authors quantified weekly caregiving hours in a prospective cohort of women and correlated this with subsequent development of BC; however, no association was found. They concluded that these specific stressors might be insufficient to trigger the immune downregulation linked to BC progression.

### Racism

We identified two studies of racism and BC risk. Taylor et al. (79) found significant relationships between major experiences of racism and BC incidence for younger women (<50 years old) in the Black Women’s Health Study, reporting a 32%-48% increased risk. Krieger et al. (80) examined the impact of legal racial discrimination (Jim Crow laws) on BC risk in US-born black and white women. The odds of being diagnosed with estrogen-receptor negative BC were slightly higher for black women born before 1965 in a Jim Crow state compared to those not born in a Jim Crow state (OR = 1.09; 95% CI: 1.06–1.13).

### Other chronic psychosocial stress themes

Several themes covered in checklists for adverse life events in epidemiological studies could turn into chronic stressors if they were repeated or endured over time. This includes financial difficulties, which, however, seem to have been studied exclusively within a life-event perspective, such as changes in financial status rather than ongoing, severe financial strain (81–83). One exception is a study included in the systematic review by Kruk et al. (68), where the authors reported positive associations with BC for a composite measure of chronic stress that included economic difficulties and interpersonal conflict, OR = 2.01; 95% CI, 1.52–2.67. The authors also reported a significant association between having a chronic disease and BC, OR = 1.85; 95% CI, 1.40–2.46 (84).

Six other studies were potentially informative about the effects of chronic psychosocial stress. Toleutay et al. (85) reported using a self-made questionnaire to measure chronic stress; however, they provided no further information on what they had measured. Surtees et al. (86) investigated chronic stress by contrasting whether participants had experienced “one or more long-term difficulties in the last 5 years” (yes, no), a very constrained measure that did not reveal any associations with BC. Four of the studies that used the LEDS referred to ongoing life difficulties, which we inspected in terms of chronic psychosocial stress. Geyer (87) reported that chronic difficulties of the highest severity occurred more often in the BC group than in the control group, but noted that he did not analyze them separately because “they are not independent of events” (p. 1545). Protheroe et al. (88) used the LEDS to capture both life events and ongoing difficulties in women with and without BC. They measured ongoing difficulties that lasted at least 4 weeks or were present at least two of the last five years prior to diagnosis. While the authors identified some ongoing difficulties in their sample, they provided no description of them, apart from stating that the occurrence of these difficulties was not associated with BC. Price et al. (89) used the LEDS to study both acute and chronic stressors, the latter defined as stressors lasting at least 6 months, and focused on exposures during the last two years prior to diagnosis, but observed no significant associations with BC.

In a recent study using the LEDS, Butow et al. (90) reported on a prospective study of 3054 women with high familial BC risk. The cohort was defined by deleterious mutations in *BRCA1/2* or other predisposition genes, or strong family history. The authors used the LEDS to identify acute and chronic stressors in 3-year intervals throughout the study but observed no associations with development of BC during a mean follow-up of 7.2 years. Butow et al. did not detail all chronic stressors, noting examples such as caring for a handicapped child and chronic neighbor disputes. They commented that their findings, focusing on high-risk women, may not generalize to the general population.

## Discussion

Preclinical research has developed and utilized an array of chronic psychosocial stress regimes, with close to 90% of studies reporting that chronic psychosocial stress invariably facilitated mammary tumorigenesis and/or metastasis in rodents. The remaining studies revealed factors that provide a more nuanced picture of the effects of chronic psychosocial stress on BC progression. One factor that modified the effects of chronic psychosocial stress was the superimposition of acute and novelty stress upon a chronic stressor, which increased neurohormonal stress responses (91) and reduced or countered the effects of the chronic stressor upon tumor development (23). Sensitive periods also were indicated, with effects of chronic stress interacting with the period for mammary gland development in puberty (25). Other factors that might impact tumor growth and metastasis include the type, duration, and severity of the stressor, the type of tumor, and a variety of host-related factors, including genetics and behavior (22, 92–95).

Do preclinical *in vivo* studies provide evidence that chronic psychosocial stress contributes to initiating BC tumor formation, rather than only tumor growth and metastasis after its initiation (37, 94)? All but one rodent study employed animal models that were BC-prone via the injection of BC cell lines or mammary tumor viruses, or through manipulated genetic or microbiological processes. The last study, however, used natural, unmanipulated Sprague-Dawley rats and reported the same effects of chronic psychosocial stress upon tumor growth as those seen in other rodent studies (34). In addition, researchers using a variety of methods and samples have reported similar overlaps between stress response pathways and mechanisms of breast carcinogenesis, suggesting that mediators of neuroendocrine stress responses affect both initiation and progression of BC (96, 97).

As noted in the introduction, systemic corticosteroid administration can reduce metastasis in estrogen-positive BC, although not in triple negative BC. Factors in addition to tumor subtype that may lead to differential effects of corticosteroid administration include hormone receptor status and tumor stage (98). One further question is whether positive corticosteroid effects in estrogen-dependent BC also could reflect that corticosteroid administration schedules are often short term. When used in human premedication schedules as adjuvant therapy before chemotherapy, corticosteroids such as Dexamethasone are typically administered for 1–3 days (99). To reduce the risk of adverse effects, including adrenal and immune system suppression, management guidelines often recommend using the lowest effective dose for the shortest duration. In rodent studies, however, corticosteroid administration schedules often last from 5 to 12 days (100, 101). One possibility is that short-term human corticosteroid administration parallels effects seen in rodent studies where an acute, novel psychosocial stressor is superimposed upon a chronic stressor - with the short-term stressor producing opposite effects to the chronic one.

Rodent studies indicated that the effects of chronic psychosocial stress on metastatic developments vary by stressor protocol. Specifically, studies report that CUMS protocols increased both primary tumor growth and metastasis, whereas social isolation protocols also increase tumor growth but less result in metastasis. Epidemiological studies did not indicate a similar differentiation, partly reflecting a lack of studies specifically on social isolation and a failure to differentiate between primary tumor growth and metastasis when defining BC study groups. While a few epidemiological studies not included in the systematic reviews analyzed the effects of poor social support - which is tangential to social isolation – on BC development, they revealed no consistent associations (89, 102–104). The preclinical indication that the metastatic effects of social isolation differ from those of chronic exposure to varied, unpredictable, and often inescapable stressors (CUMS) appears to be an untested hypothesis in human epidemiology.

Most epidemiological studies of psychosocial stress have focused on acute adverse life events. This has resulted in no robust, replicated findings, and no consensus on any etiological role of psychosocial stress in BC. The studies included in the systematic reviews and meta-analyses had only modestly focused on chronic psychosocial stress. Among the few exceptions were work stress or job strain, for which two meta-analyses were published (64, 66). Upon reflecting on the modest associations with BC observed, authors of these meta-analyses suggested that job strain may be an insufficient stressor to instigate the biological cascade of events that lead to BC development. Instead, they suggested that the effects of job strain should be considered together with the overall spectrum of stressors that women face in their lives.

Another line of epidemiological research involving chronic psychosocial stress focuses on the effects of adverse childhood experiences, which may endure over time. While a meta-analysis by Holman (65) found positive associations between adverse childhood experiences and cancer in general, results from two individual studies on BC were inconsistent (84). However, two studies reported that sexual abuse and assault in adulthood, or within ones’ lifetime, were associated with an increased BC risk, including dose-response associations (76, 77). Other studies indicate that adverse childhood experiences negatively affect women’s lives after receiving a BC diagnosis, including higher levels of depression, restricted emotional and functional well-being, reduced quality of live, and slower healing (105, 106). Among the reported consequences of adverse childhood experiences are immune alterations, such as elevated inflammatory activity, which has also been reported for women with BC exposed to childhood adversities (107). That childhood maltreatment is associated with chronic immunologic activation much later in life in BC patients is consistent with a potential role in mammary carcinogenesis.

A recent addition to epidemiological research on BC is the use of human studies to quantify allostatic load (AL). Authors of these studies have conceptualized AL as a composite physiological marker of the cumulative effects of stress upon the cardiovascular, metabolic, immunological, and neuroendocrine systems. Researchers have used a range of methods to operationalize AL, such as blood pressure, C-reactive protein, cholesterol, and triglycerids (108). In prospective studies, participants with the highest, as compared to lowest AL scores at baseline had more than double the risk of BC 10 years later (108), a 55% increased risk 11.7 years later (109), an 8% increased risk after 13.5 years (110), and 36% increased risk after 17 years (111). Other studies found that high as compared to low AL measured during the year prior to BC diagnosis was associated with more unfavorable BC clinicopathology, including increased tumor size (112, 113). However, researchers still debate what constitutes the best biological measures of AL (114). Biomarkers used as proxies to AL are likely influenced also by other factors such as poor diet, smoking, medication use, inactivity, gender, and race (115, 116). On this basis, the degree and specificity to which the AL approach has captured the effects of chronic psychosocial stress in BC appears to be unsettled. Adding measures of psychosocial stress to AL-based studies might be one step to providing clarification (117).

On the above basis, we suggest that an important reason for the lack of consensus in psychosocial epidemiological studies regarding the role of psychosocial stress in BC etiology is the limited focus on chronic psychosocial stress.

### Limitations

Several limitations bear on the validity of interpretations and conclusions in this paper.

First, we designed specific search strings to meet requirements for both precision and comprehensiveness regarding terms and expressions used in preclinical and epidemiological research. In addition, reference lists of identified papers were screened for further relevant studies. Still, this approach may have led to the omission of studies that could have been identified via broader searches.

Second, our study focused on analyzing summary data from previous systematic reviews. This implies a reliance on secondary literature. To strengthen the validity of our findings, we required that included reviews used systematic search methods and methodological quality criteria to select eligible original studies. Nevertheless, a new comprehensive meta-analysis of all prior, sufficiently sound original research in this field might provide a more precise picture.

Third, the included epidemiological studies used a variety of measures for psychosocial stress, ranging from self-made questions to validated check lists and highly structured in-depth interviews. This heterogeneity of methods may have complicated data synthesis and the ability to draw universal conclusions, requiring cautious interpretations.

### Methodological implications

Researchers analyzing conflicting evidence from epidemiological studies have focused on methodological issues, such as employing prospective rather than retrospective designs, and interviewing patients before they were given a diagnosis of BC (e.g., at mammography screenings) to prevent selective recall bias. However, improvements in study design have not produced more consistent finding (118).

Various other methodological aspects are crucial in studies that measure psychosocial stress based on participants’ reports. These include: identifying the most relevant domains and types of psychosocial stress; overcoming shortcomings in measuring stressors and quantifying stressor load; identifying relevant time intervals for exposures; evaluating interactions between different stressors – and the same stressor – over time; utilizing subjective appraisals as markers of stressor severity; and understanding the dynamics and individual variations in how stressors affect biological processes. Furthermore, while we have pointed to distinction between chronic and acute stress, these stress domains likely interact in ways that need to be captured and understood.

### Chronic stress, acute stress, and their interactions

One reason for the conflicting findings from epidemiological studies that we reviewed may be the predominant focus on acute stressors and the restricted focus on chronic stressors (4, 32). According to basic stress research, acute, point-in-time stressors with a single, short-lasting occurrence often trigger well-regulated responses in the body’s stress response systems - including the HPA axis, SAM system, and immune system - as well as in the individual’s behavior (fight, flight), which are tailored to the characteristics of the stressor (119, 120). Typically, both stress system activations and psychological alarm responses instigated by acute stress are rapidly downregulated by intrinsic recovery processes, such as negative feedback responses from the forebrain, restoring equilibrium. This may facilitate, rather than degrade, psychological growth, accomplishments, biopsychosocial and environmental adaptation, and survival (121).

In contrast, exposure to adverse social situations that endure or repeat themselves over time, such as in rodent studies with chronic stressor exposure, may be accompanied by failure of negative feedback regulation from the forebrain, and cortisol may remain elevated, together with reduced dopamine levels and cortisol metabolism (122). This may slowly drain an individual’s resources, leading the nervous system and body toward an exhaustion stage where cells cannot maintain normal functions, increasing the risk of negative health effects (123, 124). Therefore, stressful exposures that persist despite efforts to avoid or ameliorate them typically have stronger health effects than acute stressors and can be considered as potent predictors of health (125–128).

While acute stressful events usually do not have similarly persistent consequences as chronic stress, severe acute stressors may lead to prolonged responses – such as post-traumatic stress disorder (PTSD) in 5-10% of exposed individuals. When exposure to an intense stressor is more extensive, the percentage with prolonged responses increases up to 30% (129, 130).

One mechanism in prolonged responses may be the degree to which an exposure leads to sustained appraisals of threat and danger. Still, research has yet to discern which qualitative features of sustained post-acute stress appraisals most potently impact biological mechanisms associated with negative health sequelae, including BC. For example, while acute stressors such as death of a loved one and divorce may leave an enduring impact and be life-changing, subsequent negative health effects might depend on whether the event was accompanied by appraisals of threat and danger, with feelings of alarm and fear, rather than by feelings of sadness and sorrow or even by relief (129).

Furthermore, individuals may adapt and habituate both psychologically and biologically to stressors that are repeated or endure over time, which in turn may depend on stressor characteristics and the individual’s subjective appraisals, as well as coping style, social networks. and more. The existing gap in understanding the relevant distinctions between acute and chronic stress, and the transitional phase between them, is a caveat in current research into the stress–health relationship (131).

While chronic stress could have a primary role in the stress-health association, it may be important to assess acute stressful events in the context of chronic stress. Various interactions between chronic and acute stress have been reported. Norris and Uhl (132) observed that chronic stress mediated long-term effects of an acute stressor (a hurricane) on psychological distress. Other studies indicate that chronic stress increases an individual’s physiological and psychological susceptibility to acute stressful events (133, 134), as conceptualized in stress proliferation theories (135). McGonagle and Kessler conjectured that chronic stress may also reduce susceptibility to subsequent acute stressors (127). Similar views are reflected in the stealing hypothesis, maturation hypothesis, and crisis theory, which suggest that moderate temporary stress exposures may increase coping and reduce negative effects of subsequent stressors on health (136). Moreover, as noted above, preclinical rodent studies have reported significant interaction effects of acute and novelty stress upon an ongoing chronic psychosocial stressor (23, 24), supporting the view that both stressor types and their interactions should be measured.

### Types of chronic psychosocial stress

A wide range of chronic psychosocial stressors might be relevant to BC development, ranging from social-level factors, such as enduring financial burden and discrimination, to intimate relational level factors, such as partner stress. Social exposome theory emphasizes social interactions as the most proximate sources of psychosocial exposures, acting as filters through which the broader societal context – including socioeconomic, cultural, and political characteristics - reaches individuals (137). This multidimensional approach aims to integrate all relevant social and psychosocial exposures into a holistic perspective, understanding how the entirety of individual and structural social exposures translates into health outcomes. Researchers within this framework model the co-occurrences and reciprocities of stressors within and across levels, the duration of individual stressors, overall stressor landscapes, and their embodiment as health effects (137).

Other theories explicitly target the relational perspective, such as the “linked lives” approach (138), which suggests that people who live together in intimate relational contexts over time (typically spouses) are closely tied and mutually influential, both emotionally and behaviorally, at the dyadic level. People in close partnerships are seen as interdependent in their experiences of and reactions to stress, including contemporaneous associations and cross-over or partner effects (139). In our context, when a woman’s intimate partner experiences chronic psychosocial stress, the partner’s stress may become a major source of stress for her, with long-term consequences for her mental and physical health.

### Subjective appraisals

A likely shortcoming in human BC research is that most studies have not included the subjective dimension of stress appraisals. As pointed out by Lazarus and Folkman (140), it is neither the individual nor the environment alone that produces stress, but a complex transaction between the two, in which the individual’s subjective appraisals are central to whether environmental exposures exceed one’s coping resources. Since the pioneering research by Selye in the 1930s, it has been apparent that physiological responses (e.g. pituitary gland activation) to stressors are tightly linked to the individual’s psychological, subjective appraisals of the stressors; they are not a reflection of objective stressors impinging directly upon the body. Subjective distress and fear are intricate parts of traumatizing stress that occur because of perceived threats to one’s mental or physical wellbeing, which in turn are accompanied by the above noted cascade of biological responses (141). Subjective distress severity levels may predict negative health outcomes better than stressor exposure as such (142) and may be what adds weight to different stressors in terms of (individual) stressor severity (143) and should be included in etiological studies. The role of subjective appraisals also has been highlighted by BC researchers, with Nielsen and Grønbæk (56) commenting that a person’s appraisal of psychosocial stress could be just as crucial for BC onset as stressor exposures per se.

Several epidemiological studies have utilized external judges to rate participants’ subjective distress levels following exposure to stressors based on standardized norms. Although this approach is common in studies employing checklists and the LEDS instrument, it poses a significant challenge: it fails to capture individual variations in distress, which can be considerable for any given adverse event – a limitation known as intracategorical variability (144) or the reality principle (145). If subjective distress is indeed the most pertinent measure of a stressor’s severity and its bodily impact (142), then relying on general norms is theoretically problematic and warrants reconsideration.

### Exposure period for breast cancer development

The process of BC development is often estimated to occur over 15 to 20 years (146–148). This contrasts with the most common period covered in retrospective studies of psychosocial stress in human BC, which is often restricted to the last 1–5 years prior to diagnosis (although prospective studies usually have longer timeframes). It is possible that constraining the measurement period to the last few years before diagnosis mostly captures the period of aberrant cell division and tumor growth, while etiologically relevant stressor exposures associated with BC initiation take place earlier. Other evidence suggests that the relevant exposure period spans several decades; for example, early menarche (prior to age 12) is a known causal factor in BC. Furthermore, it is well established that stressor exposure during upbringing may sensitize and biologically prime the individual to exhibit altered biopsychobehavioral responses to stress later in life (149).

In line with this, rodent research indicates that predispositions toward cancer-initiating events may be established early in life. One example is Boyd et al. (36), who reported that chronic, moderate early-life stress - in the form of prolonged maternal separation in the first weeks of life - caused morphologic and molecular changes in the mammary glands in adulthood, resulting in a higher incidence of carcinogen-induced mammary tumors. In line with authors in other health fields (16, 150), we suggest that the relevant exposure period to cover in epidemiological psychosocial studies of chronic psychosocial stress in BC etiology is the entire life-course.

### Implementing the study design elements – an example

A life-course perspective on how genetic, biological, and psychosocial factors shape health trajectories from birth to old age and death has been advocated by bodies such as the WHO and UN, as well as by the subfield of life-course epidemiology (151, 152). Life-course theories emphasize the importance of temporality and contextuality in understanding how individual experiences evolve over time, along with the interconnectedness of life phases, psychosocial exposures, and interpersonal relationships. These approaches have delineated several models on how co-occurring and successive stressor encounters over the course of life affect health. Central models are (i) the sensitive period model, which focuses on the differential effects of exposures depending on their timing, (ii) the accumulation model, which considers summary effects of concomitant and successive exposures and their duration, and (iii) the pathway, chain-of-risk, or diathesis-stress model, which sequentially links multiple stress exposures and other potentially causal factors, such as how genetics or early stress exposures biologically program the structure or function of organs, tissues, or body systems, thereby influencing effects of stress exposures later in life (152, 153).

A central measurement approach in life-course models is lifetime history calendars (LHC) (154). An LHC can be visualized as a two-dimensional grid or table, with three key characteristics; (a) a graphical display of the time dimension on the X-axis, with the reference period divided into smaller time units (e.g. years); (b) the graphical display encompasses several themes and stress domains entered as rows along the Y-axis; and (c) the interview starts with eliciting information on personal landmark events such as marriages, births, and changes in geographical residence, which function as reference points for recalling details in the thematic areas that subsequently will be probed (155). The interviewer works through the thematic areas and fills in the cells of the matrix with information provided by the respondent. LHC designs help respondents visually and mentally relate the timing of several kinds of life experiences to each other, encouraging top-down and parallel retrieval, with the calendar display aiding attention to any inconsistencies in the timing of exposures between different domains. LHCs are used in both cross-sectional, retrospective, and prospective studies (156) and have been validated against traditional questionnaire approaches (157–161).

**Figure 4:**
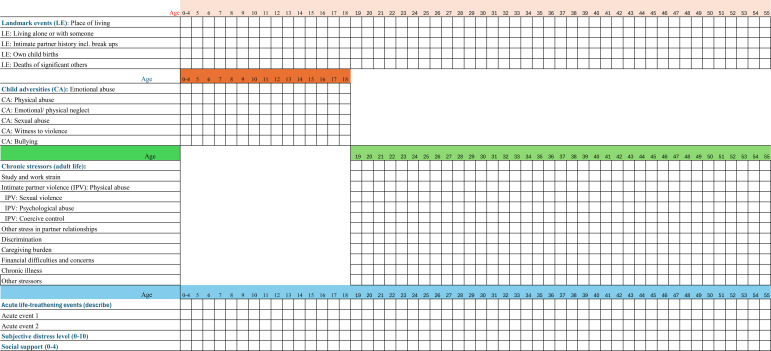
Example of life history calendar instrument.

An example of an LHC set up to guide investigations into chronic psychosocial stress in BC using a life-course perspective is illustrated in **Figure 4**. The example assumes a retrospective design for women enrolled at age 55, with a temporal resolution of one year. The variables include a set of landmark events, followed by exposure to psychosocial stress in childhood and adulthood, acute life-threatening stressors at any time, and subjective distress level, in addition to a possible covariate (social support) (**Table 3**).

**Table 3:** Example of variables in a life history calendar (LHC) to investigate exposure to psychosocial stress, with focus on chronic stressors.

Type of variables	Content	Measurement approach
Landmark events	Place of living/moving; Living alone or with someone; Intimate partner history including break ups; Own child births; Deaths of significant others	Simple questions (e.g. “where did you live?”, “tell me about your partner history”)
Childhood psychosocial stress	Emotional abuse by parents/other caregivers; Physical abuse by parents/other caregivers; Emotional or physical neglect/care failure - by parents/other caregivers; Sexual abuse by caregivers or other adults; Witness to violence against others; Bullying verbally or physically by peers	Short versions of a validated questionnaire for exposure to adversities during childhood and adolescence and that include timing information, e.g. Maltreatment and abuse chronology of exposure scale
Chronic stressor exposures in adult life	Study and work strain	Accommodate simple questions based on valid questionnaires, or describe each variable with covering examples
	Intimate partner violence (IPV; any type)	
	Discrimination	
	Caregiver burden	
	Financial difficulties and concerns	
	Chronic illness	
Acute life-threatening stressors	Any type identified by respondent	Question about occurrence and type of stressor, examples may be provided (e.g. a list)
Subjective distress level	Subjective distress level	Subjective units of distress scale, or a scale to measure fear
Social support	Social support	Accommodate a simple question

In the example, we suggest using validated screening instruments for childhood exposures (e.g (162).) and single questions to assess each exposure type in adult life, preferably derived from established instruments. To assess financial difficulties, for example, a question adapted from a prior life-course survey (163) is: “​During that time (or that year), how hard was it for you to pay for the very basics like food, housing, clothing, and heating?” (1 = not very hard, 2 = somewhat hard, 3 = hard, or 4 = very hard). Measuring intimate partner violence could be achieved by using descriptive words for each main subdimension of the variable (physical abuse, sexual violence, psychological abuse, and coercive control) (164). Literature supports describing psychological abuse to participants as, for example, harassment, verbal abuse (name calling, degradation, insults, blaming, intimidation, threats, stalking, humiliation), or deprivation of necessities like food, money, transportation, or access to health care.

In the overall LHC scheme, a special challenge relates to measuring participants’ subjective, experiential level of distress annually. One approach utilizes a one-dimensional scale for distress severity, such as the Subjective Unit of Distress Scale (SUDS). This scale has been validated in a variety of contexts and used both for clinical assessments and as outcome measure in therapy, with one example being the Distress Thermometer used in the cancer field (165). Using SUDS, the distress level is typically scored on a visual analogue scale ranging from 0 (completely calm) to 10 (highest distress possible) (166). However, as distress is a broad term, an alternative approach could target the specific emotions associated with appraising a stressor as threatening. One candidate is fear, which, although often short-lived, can focus on perceived threats, such as an abusive partner not being physically present.

Regarding important covariables, we added social support in this example. A parsimonious way to assess social support is to combine two of the three items from the Oslo support scale (OSS3) (167) into one question: “Did you have people close to you who were interested, showed concern, and who you could count on in times of personal problems?”. The response is scored from 0 (not at all/never) to 4 (fully/always).

In prospective study designs, LHCs first may initially be used retrospectively at baseline to cover stress exposures from birth until the present (e.g. for women aged 30-35), and subsequently at each subsequent measurement point to cover the interval since the last assessment.

Because LHC designs in BC research are novel, an initial phase is necessary to: (1) investigate instrument reliability and validity; (2) operationalize conceptual models; (3) develop summary scores for psychosocial stress; (4) establish norms; and (5) delineate statistical approaches (168).

## Conclusions

Preclinical rodent studies, alongside a subset of human epidemiological studies, suggest that chronic psychosocial stress is a relevant risk factor in BC development. The current lack of consensus in mainstream BC epidemiological research may reflect a predominant focus on short-term stressors rather than chronic psychosocial stress. We suggest that explicitly targeting chronic psychosocial stress – specifically by querying participants about longitudinal life experiences – would deepen the understanding of BC etiology. A life-course perspective, focusing on the duration of chronic stressor exposures, concurrent/temporal interactions with other chronic stressors and acute stressors, and measures of severity based on individual subjective appraisals, should be utilized to further investigate these risks.

## Data Availability

The original contributions presented in the study are included in the article/supplementary material. Further inquiries can be directed to the corresponding author.
